# 522 Testosterone Supplementation in Patients with Burn Hypermetabolism

**DOI:** 10.1093/jbcr/irae036.157

**Published:** 2024-04-17

**Authors:** Alexandra DeWitt, Anastasiya Ivanko, Jonathan E Schoen, Aimee Keating, Herb A Phelan, Jeffrey E Carter

**Affiliations:** University Medical Center New Orleans, New Orleans, LA; University Medical Center (LSU Health), New Orleans, LA; University Medical Center New Orleans, Metairie, LA; Louisiana State University Health Sciences Center- New Orleans, New Orleans, LA; University Medical Center New Orleans, New Orleans, LA; University Medical Center (LSU Health), New Orleans, LA; University Medical Center New Orleans, Metairie, LA; Louisiana State University Health Sciences Center- New Orleans, New Orleans, LA; University Medical Center New Orleans, New Orleans, LA; University Medical Center (LSU Health), New Orleans, LA; University Medical Center New Orleans, Metairie, LA; Louisiana State University Health Sciences Center- New Orleans, New Orleans, LA; University Medical Center New Orleans, New Orleans, LA; University Medical Center (LSU Health), New Orleans, LA; University Medical Center New Orleans, Metairie, LA; Louisiana State University Health Sciences Center- New Orleans, New Orleans, LA; University Medical Center New Orleans, New Orleans, LA; University Medical Center (LSU Health), New Orleans, LA; University Medical Center New Orleans, Metairie, LA; Louisiana State University Health Sciences Center- New Orleans, New Orleans, LA; University Medical Center New Orleans, New Orleans, LA; University Medical Center (LSU Health), New Orleans, LA; University Medical Center New Orleans, Metairie, LA; Louisiana State University Health Sciences Center- New Orleans, New Orleans, LA

## Abstract

**Introduction:**

After major burn injuries, patients experience a hypermetabolic response that leads to hyperdynamic circulatory, physiologic, catabolic, and immune system responses. Many transdisciplinary approaches have been tested to mitigate this postburn hypermetabolic syndrome such as comprehensive burn rehabilitation, nutritional support, monitoring, and pharmacological interventions. Oxandrolone, a synthetic analog of testosterone, has been shown to have higher anabolic properties and minimal androgenic effects and studied intensely in burn care. In 2023, the FDA asked all manufacturers of oxandrolone to cease production and subsequently removed the drug from market on June 28, 2023. Our study examines the cost and operationalization of an intramuscular (IM) testosterone protocol as an alternative in male burn patients with ≥20% total body surface area (TBSA).

**Methods:**

A cost analysis was conducted using the National Drug Codes (NDC) and commercially available benchmarking data. The cost of oxandrolone and testosterone were included in our analysis. Following a thorough literature review, a protocol was developed for adult male patients with >20% TBSA burn injuries. Patients undergoing gender affirming hormone therapy, untreated obstructive sleep apnea, or hormonal blockers for cancer were excluded from the protocol.

**Results:**

The protocol begins with total serum testosterone levels at the time of admission and weekly thereafter for male patients with ≥20% TBSA injury. Once a testosterone level < 150 ng/dL is detected, supplementation of testosterone with 200 mg IM once weekly for two weeks is started. Weekly evaluations include total body weight, metabolic panels, and nutritional panels. Cost comparison between oxandrolone and testosterone show an equivalent unit cost of $8.62 versus $8.38, respectively.

**Conclusions:**

Drug shortages and supply chain challenges are becoming more common in burn care. We hypothesize testosterone to be both a clinically effective and cost-effective alternative to oxandrolone in the setting of burn hypermetabolism in male patients with major burns. Currently, burn experts are working with endocrinologists to assess pharmacologic alternatives for female patients and have obtained IRB-approval to evaluate both safety and efficacy of testosterone supplementation in the setting of burn hypermetabolism.

**Applicability of Research to Practice:**

This study evaluates the safety and efficacy of testosterone supplementation as an alternative to oxandrolone in the setting of burn hypermetabolism. This study helps provide guidance to other burn centers in filling the gap in therapy that now exists in the pharmacologic management of hypermetabolic syndrome in patients with severe burns.

